# Assisting the Diagnosis of Cirrhosis in Chronic Hepatitis C Patients Based on Machine Learning Algorithms: A Novel Non‐Invasive Approach

**DOI:** 10.1002/jcla.70054

**Published:** 2025-05-19

**Authors:** Emre Dirican, Tayibe Bal, Yusuf Onlen, Figen Sarigul, Ulku User, Nagehan Didem Sari, Behice Kurtaran, Ebubekir Senates, Alper Gunduz, Esra Zerdali, Hasan Karsen, Ayse Batirel, Ridvan Karaali, Hatice Rahmet Guner, Tansu Yamazhan, Sukran Kose, Nurettin Erben, Nevin Koc Ince, Iftihar Koksal, Nefise Oztoprak, Gulsen Yoruk, Suheyla Komur, Sibel Yildiz Kaya, Ilkay Bozkurt, Ozgur Gunal, Ilknur Esen Yildiz, Dilara Inan, Sener Barut, Mustafa Namiduru, Selma Tosun, Kamuran Turker, Alper Sener, Kenan Hizel, Nurcan Baykam, Fazilet Duygu, Hurrem Bodur, Guray Can, Hanefi Cem Gul, Ayse Sagmak Tartar, Guven Celebi, Mahmut Sunnetcioglu, Oguz Karabay, Hayat Kumbasar Karaosmanoglu, Fatma Sirmatel, Omer Fehmi Tabak

**Affiliations:** ^1^ Department of Biostatistics Faculty of Medicine, Hatay Mustafa Kemal University Hatay Turkey; ^2^ Department of Infectious Diseases and Clinical Microbiology Faculty of Medicine, Bolu Abant Izzet Baysal University Bolu Turkey; ^3^ Department of Infectious Diseases and Clinical Microbiology Faculty of Medicine, Akdeniz University Antalya Turkey; ^4^ Department of Infectious Diseases and Clinical Microbiology Health Sciences University, Istanbul Training and Research Hospital Istanbul Turkey; ^5^ Department of Infectious Diseases and Clinical Microbiology Faculty of Medicine, Cukurova University Adana Turkey; ^6^ Department of Gastroenterology Medicana International Istanbul Hospital Istanbul Turkey; ^7^ Department of Infectious Diseases and Clinical Microbiology Health Sciences University, Sisli Hamidiye Etfal Training and Research Hospital Istanbul Turkey; ^8^ Department of Infectious Diseases and Clinical Microbiology Health Sciences University, Haseki Training and Research Hospital Istanbul Turkey; ^9^ Department of Infectious Diseases and Clinical Microbiology Faculty of Medicine, Harran University Sanlıurfa Turkey; ^10^ Department of Infectious Diseases and Clinical Microbiology Health Sciences University, Dr. Lutfi Kirdar Kartal Training and Research Hospital Istanbul Turkey; ^11^ Department of Infectious Diseases and Clinical Microbiology Cerrahpasa Faculty of Medicine, Istanbul University‐Cerrahpasa Istanbul Turkey; ^12^ Department of Infectious Diseases and Clinical Microbiology Faculty of Medicine, Ankara Yildirim Beyazit University, Ankara City Hospital Ankara Turkey; ^13^ Department of Infectious Diseases and Clinical Microbiology Faculty of Medicine, Ege University Izmir Turkey; ^14^ Department of Infectious Diseases and Clinical Microbiology Faculty of Medicine, Dokuz Eylül University Izmir Turkey; ^15^ Department of Infectious Diseases and Clinical Microbiology Faculty of Medicine, Eskisehir Osmangazi University Eskisehir Turkey; ^16^ Department of Infectious Diseases and Clinical Microbiology Faculty of Medicine, Duzce University Duzce Turkey; ^17^ Department of Infectious Diseases and Clinical Microbiology Acibadem University, Atakent Hospital Istanbul Turkey; ^18^ Department of Infectious Diseases and Clinical Microbiology Cerrahpasa Faculty of Medicine, Istanbul University‐Cerrahpasa Corum Turkey; ^19^ Department of Infectious Diseases and Clinical Microbiology Faculty of Medicine, Ondokuz Mayis University Samsun Turkey; ^20^ Department of Infectious Diseases and Clinical Microbiology Faculty of Medicine, Samsun University Samsun Turkey; ^21^ Department of Infectious Diseases and Clinical Microbiology Faculty of Medicine, Recep Tayyip Erdoğan University Rize Turkey; ^22^ Department of Infectious Diseases and Clinical Microbiology Faculty of Medicine, Gaziantep University Gaziantep Turkey; ^23^ Department of Infectious Diseases and Clinical Microbiology Health Sciences University, Bozyaka Training and Research Hospital Izmir Turkey; ^24^ Department of Infectious Diseases and Clinical Microbiology Health Sciences University, Bağcılar Training and Research Hospital Istanbul Turkey; ^25^ Department of Infectious Diseases and Clinical Microbiology Health Sciences University, Izmir Katip Celebi Training and Research Hospital Izmir Turkey; ^26^ Department of Infectious Diseases and Clinical Microbiology Faculty of Medicine, Gazi University Ankara Turkey; ^27^ Department of Infectious Diseases and Clinical Microbiology Faculty of Medicine, Hitit University Corum Turkey; ^28^ Department of Infectious Diseases and Clinical Microbiology Faculty of Medicine, Gaziosmanpaşa University Tokat Germany; ^29^ Department of Infectious Diseases and Clinical Microbiology Health Sciences University, Ankara City Hospital Ankara Turkey; ^30^ Department of Gastroenterology Faculty of Medicine, Abant Izzet Baysal University Bolu Turkey; ^31^ Department of Infectious Diseases and Clinical Microbiology Health Sciences University, Gülhane Training and Research Hospital Ankara Turkey; ^32^ Department of Infectious Diseases and Clinical Microbiology Faculty of Medicine, Fırat University Elazıg Turkey; ^33^ Department of Infectious Diseases and Clinical Microbiology Faculty of Medicine, Zonguldak Bulent Ecevit University Zonguldak Turkey; ^34^ Department of Infectious Diseases and Clinical Microbiology Faculty of Medicine, Sakarya University Sakarya Turkey; ^35^ Department of Infectious Diseases and Clinical Microbiology Health Sciences University, Bakırköy Dr. Sadi Konuk Training and Research Hospital Istanbul Turkey; ^36^ Department of Internal Medicine Division of Infectious Diseases Izmir Tınaztepe University Faculty of Medicine İzmir Turkey

**Keywords:** alfa‐feto protein, chronic hepatitis C, classification, diagnosis of cirrhosis, machine learning

## Abstract

**Aim:**

This study aimed to determine the important features and cut‐off values after demonstrating the detectability of cirrhosis using routine laboratory test results of chronic hepatitis C (CHC) patients in machine learning (ML) algorithms.

**Methods:**

This retrospective multicenter (37 referral centers) study included the data obtained from the Hepatitis C Turkey registry of 1164 patients with biopsy‐proven CHC. Three different ML algorithms were used to classify the presence/absence of cirrhosis with the determined features.

**Results:**

The highest performance in the prediction of cirrhosis (Accuracy = 0.89, AUC = 0.87) was obtained from the Random Forest (RF) method. The five most important features that contributed to the classification were platelet, αlpha‐feto protein (AFP), age, gamma‐glutamyl transferase (GGT), and prothrombin time (PT). The cut‐off values of these features were obtained as platelet < 182.000/mm^3^, AFP > 5.49 ng/mL, age > 52 years, GGT > 39.9 U/L, and PT > 12.35 s. Using cut‐off values, the risk coefficients were AOR = 4.82 for platelet, AOR = 3.49 for AFP, AOR = 4.32 for age, AOR = 3.04 for GGT, and AOR = 2.20 for PT.

**Conclusion:**

These findings indicated that the RF‐based ML algorithm could classify cirrhosis with high accuracy. Thus, crucial features and cut‐off values for physicians in the detection of cirrhosis were determined. In addition, although AFP is not included in non‐invasive indexes, it had a remarkable contribution in predicting cirrhosis.

**Trial Registration:**

Clinicaltrials.gov identifier: NCT03145844

## Graphical abstract

Using the results of routine laboratory tests, it was proved by machine learning that cirrhosis can be diagnosed at a high rate. Threshold values were determined for the diagnosis of cirrhosis.

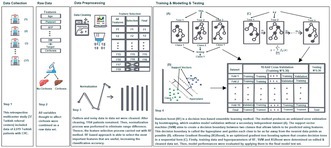

## 1 Introduction

It is estimated that approximately 58 million of the global population is infected with hepatitis C virus (HCV) [1]. After exposure to HCV, about 55%–85% of patients progress to chronic hepatitis C (CHC) infection, which leads to liver fibrosis secondary to liver inflammation. Over time, the progression of liver fibrosis leads to cirrhosis, which may then progress to end‐stage liver disease and hepatocellular carcinoma (HCC). Therefore, accurate detection of cirrhosis is not only important for the selection of the optimal direct‐acting antiviral (DAA) regimen and its duration but also helps in the decision of whether or not to perform surveillance for HCC and endoscopy for esophageal varices after therapy [2, 3, 4]. Although liver biopsy is the gold standard for the diagnosis of cirrhosis, its high cost, invasive nature with risk of complications, the heterogeneous distribution of hepatic fibrosis, and sampling and/or pathological interpretation variabilities are the most important limitations of liver biopsy in predicting cirrhosis. Therefore, research efforts to find alternative, cheaper, practical, and safe non‐invasive methods that can predict the diagnosis of cirrhosis have led to the development of non‐invasive tests [5].

It is still controversial whether non‐invasive methods can replace liver biopsy in the prediction of the presence of cirrhosis [6]. However, the current recommendation of the European Association for the Study of the Liver (EASL) is that non‐invasive tests can be used as an alternative to liver biopsy in the evaluation of the presence of cirrhosis before DAA therapy, to determine the duration of treatment and the DAA antiviral therapy to be used in the treatment. It is expected that at least two different non‐invasive methods will be used together in the evaluation, and the results of these two methods will be consistent with each other [7]. However, this recommendation is difficult to implement because non‐invasive alternatives such as fibroscan and elastography are expensive and are not available in every center due to the need for personnel trained in their application, which can be done together with scores such as the aspartate aminotransferase (AST)‐to‐ platelet ratio index (APRI) and the Fibrosis‐4 (FIB‐4) index calculated using routine laboratory parameters [8]. Therefore, there is a need for new non‐invasive alternatives to be developed for the detection of cirrhosis.

Machine learning (ML) algorithms have good forecasting capabilities for the prediction of various diseases, which has led to the increased use of these algorithms in the field of health care [9]. However, studies [5, 10, 11, 12, 13, 14] on cirrhosis prediction did not provide information about the treatment regimen or could not contribute significantly to the clinician's opinion. However, ensuring the reliability of non‐invasive ML algorithms for cirrhosis detection may require a specific focus on the pre‐treatment cohort, as this approach helps minimize the variability introduced by post‐treatment physiological changes.

To the best of our knowledge, no previous study has examined CHC patients before starting DAA therapy, so it is unclear if the previous models can be transferred to CHC patients who will receive DAA therapy. Therefore, this study aimed to use routine laboratory test results in ML algorithms to reveal the classifiability/determinability of cirrhosis in CHC patients prior to DAA therapy, and after that to determine the important features in this diagnosis and their cut‐off values.

## 2 Methods

ML algorithms are trained using a known data set (“supervised learning”), which is then applied to the test data for prediction. The whole process from the creation of the raw data set to the testing of the trained model is depicted in Figure 2.

### 2.1 Patients and Data

This retrospective multicenter study used data obtained from the HEP‐C TURKEY database, which is a multicenter, observational registry of clinical data collected from 2619 Turkish patients with CHC with real‐world experience of DAA therapy between April 2017 and December 2019. It is registered by The Clinical Microbiology Specialty Society (EKMUD) and The Viral Hepatitis Society (VHSD) Infectious Diseases of Turkey, including data from 37 Turkish referral centers.

The inclusion criteria for this study were: age > 18 years, positive for both HCV antibodies and HCV RNA, a history of liver biopsy with available METAVIR score, and laboratory results performed at the same time as the liver biopsy. Exclusion criteria were defined as follows: known co‐infection with hepatitis B virus (HBV) or human immunodeficiency virus (HIV), chronic alcoholism, immunosuppression or malignancy, or patients under DAA therapy.

**FIGURE 1:**
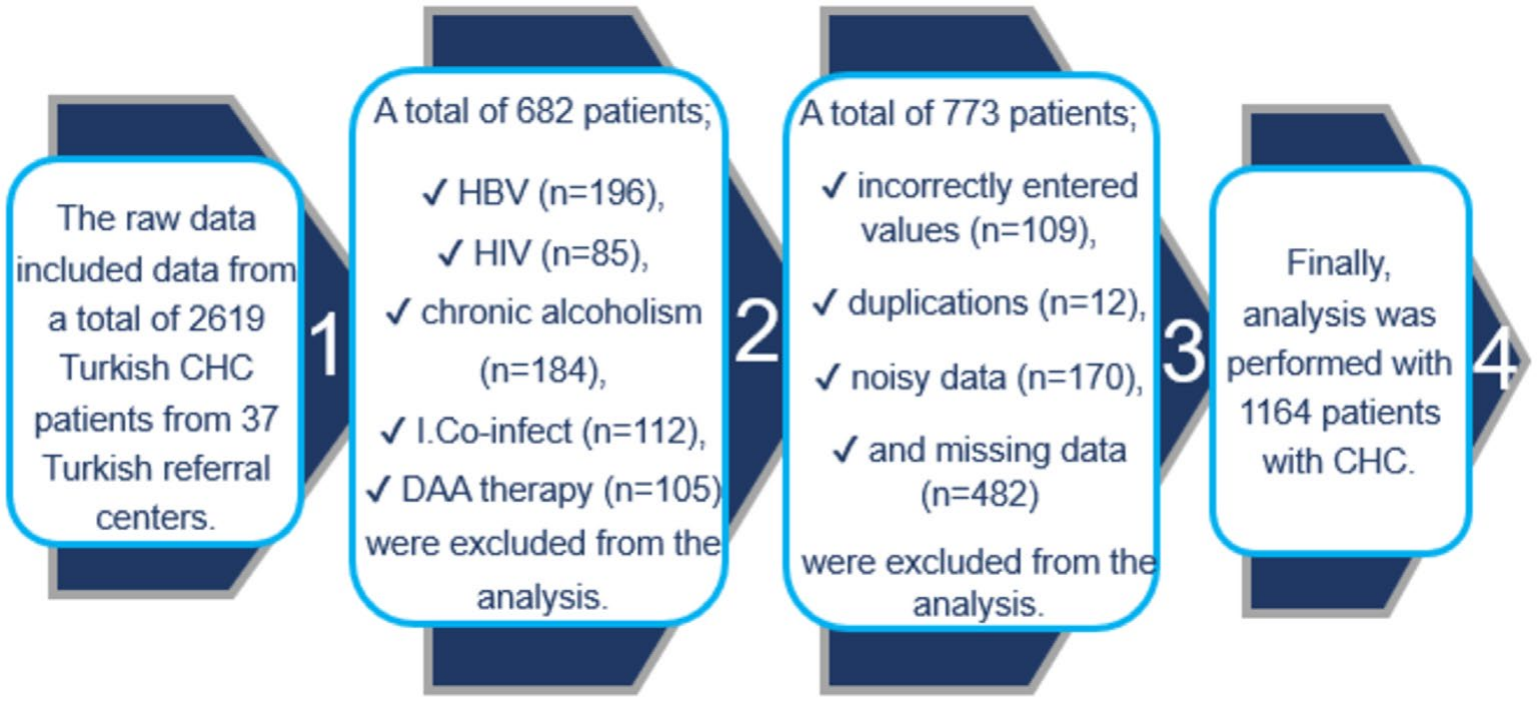
Flow chart; process of counting data until ready for final analysis, I. Co‐infect: Immunosuppression or co‐infection with malignancy.

Prior to data cleansing, 1937 patients met the inclusion criteria (Figure 1). Demographic, clinical, histopathological, and laboratory data were collected via the web‐based reporting platform. Input features were age, gender (male, female), body mass index (BMI), comorbidities, coronary artery disease (CAD), hypertension, diabetes mellitus (DM), and the results of laboratory tests performed before DAA therapy, including platelet, complete blood count analysis (CBC), serum alanine aminotransferase (ALT), aspartate aminotransferase (AST), alkaline phosphatase (ALP), gamma‐glutamyl transpeptidase (GGT), total bilirubin (TBIL), prothrombin time (PT), albumin, alpha‐fetoprotein (AFP), HCV genotype, and HCV RNA.

**FIGURE 2:**
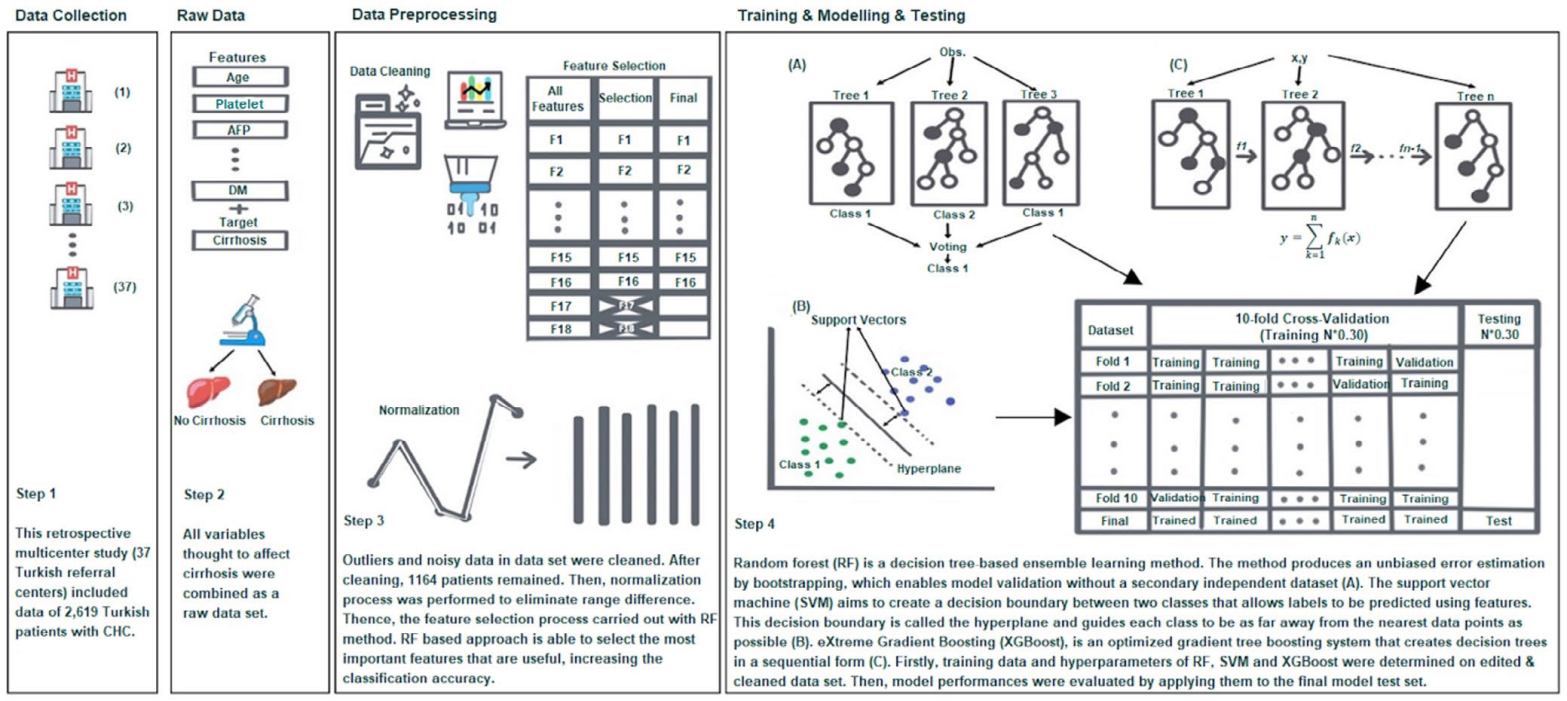
The entire ML process, from raw data set to prediction.

The stage of liver fibrosis was determined from F0 to F4, based on the METAVIR score [15]. The target variable (binary) was the status at which cirrhosis was classified: no cirrhosis (F0–F3) and cirrhosis (F4). Patients were identified as having cirrhosis if they had a liver biopsy showing cirrhosis (Metavir F4) (Figure 2).

### 2.2 APRI and FIB‐4 Scores Calculation

The aspartate aminotransferase to platelet ratio index (APRI) and the Fibrosis 4 score (FIB‐4) are non‐invasive alternatives to liver biopsy to detect liver fibrosis stages or the presence of cirrhosis. The upper limit of normal (ULN) of AST was 40 IU/L. APRI scores were calculated using the following formula: APRI score = [(AST/ULN AST) × 100]/platelet (10^9^/L) [16]. FIB‐4 scores were calculated as: FIB‐4 = Age ([year] × AST [U/L])/((platelet [10 (9)/L]) × (ALT [U/L]) (1/2)) [17].

### 2.3 Data Preprocessing

#### 2.3.1 Data Cleansing

Both box plots and clinician opinions were taken into account when removing outliers. Observations that were determined to be extreme in the graph or not compatible with the laboratory test result according to the clinician's approach were excluded. In addition, the data set was organized by removing noisy data.

#### 2.3.2 Standardization

Min‐max normalization was used so that the variation caused by features with substantially different reference ranges did not have a negative effect on the classification.

### 2.4 Methodology

#### 2.4.1 Feature Selection

Random Forests (RF) were used to select the most significant features that contributed to distinguishing cirrhosis status (present‐absent). The reason for using RF was that it increased the classification accuracy while reducing the features and was a reliable method for selection in experimental results [18].

**FIGURE 3:**
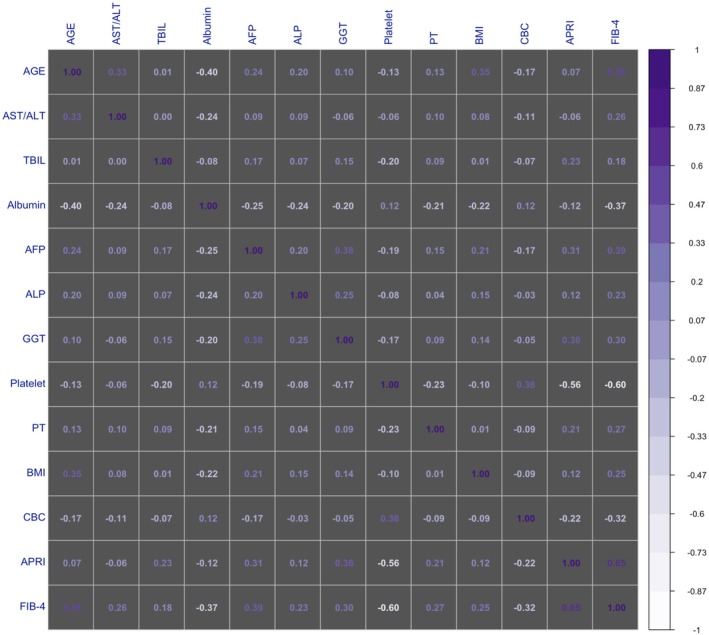
The correlogram expresses the correlation consisting of only continuous features in the data set.

The analysis was started with 18 features. Genotype and HCV‐RNA were excluded from the data set as they did not contribute to the classification after the selection process. The present study was conducted with 16 features. Correlations between features were also evaluated for the data set in which categorical features were excluded. There was no correlation value above 0.60 between features (Figure 3) [19].

#### 2.4.2 Training–Testing

The training data was 70% (816 obs.) of the cleaned and standardized data set, and the remaining 30% (348 obs.) was used for the test set. Optimum values for hyperparameters of ML classifier algorithms were obtained using grid search and 10‐fold cross‐validation.

#### 2.4.3 Classification Methods and Cut‐Off Values

Classification is an ML approach that is used to forecast group membership for data sets [20]. Three different ML methods, namely, RF, Support Vector Machines (SVM) linear and radial‐based, and eXtreme Gradient Boosting (XGBoost) were used to classify cirrhosis according to the determined features. The RF method has the ability to classify non‐parametric structures with high performance and to determine the variable importance [21]. Another ML technique, SVM, is generally much faster and is known to have high performance for large data sets [22]. Known as a kind of gradient boosting machine, XGBoost was developed mainly in two aspects: to speed up tree construction and to propose a new distributed algorithm for tree search. With these features, it can provide very successful results for classification tasks [23]. Different methods were used to determine cut‐off values of the important features. In determining these values, univariate ROC analysis results, clinician opinions, the cut‐off value providing the highest information according to the gini index in the RF model final tree, and the Multivariate Adaptive Regression Spline (MARS) results were taken into account. The logistic regression model was used to calculate the odds ratio for the cirrhosis risk of the cut‐off values together with all the other features.

#### 2.4.4 Evaluating the Models

A confusion matrix was created to evaluate the performance of ML techniques. Accuracy, sensitivity, specificity, and positive and negative predictive values were calculated from this matrix, as well as the area under the curve (AUC) and confidence intervals with software [24].

#### 2.4.5 Statistical Analysis

Data obtained in the study were analyzed statistically using SPSS version 25 software (Armonk, NY: IBM Corp.). Descriptive statistics of the features were stated as median and percentile values (25th—75th), frequency, and percentage. Mann Whitney U test was applied for numerical comparison of the groups. The performance of the APRI and FIB‐4 scores was compared with ML models using several metrics. Brier score and AUC were used for overall performance. A lower Brier score indicates a better model fit, with values closer to 0 reflecting better prediction accuracy. The closer the AUC is to 1, the better the model performs. Calibration was assessed using the Hosmer–Lemeshow test [25], where a *p*‐value > 0.05 suggests good calibration, and values below 0.05 suggest poor calibration. For reclassification, Net Reclassification Index (NRI) and Integrated Discrimination Improvement (IDI) were calculated with 95% confidence intervals. Both NRI and IDI values increase with better model performance, where higher values indicate better reclassification and discrimination ability [26, 27]. Finally, Decision Curve Analysis (DCA) was conducted to assess the clinical usefulness of the models. A higher net benefit across a range of threshold probabilities indicates a model with better clinical utility [28]. In addition, hyperparameter tuning; the range of values for each hyperparameter was determined based on prior literature and exploratory experiments. We employed grid search to explore different hyperparameter combinations. A 3‐repeated 10‐fold cross‐validation approach was used during tuning to ensure robustness and minimize overfitting. The selection of optimal values was based on overall model performance across multiple metrics (AUC, accuracy, sensitivity, specificity, and others as relevant), ensuring a well‐balanced and generalizable model. Imputation; there were no missing values in the dataset; therefore, imputation was not required.

R Studio version 0.92.382 was used for all ML techniques with the “caret v6.0–90,” “corrplot v0.92,” “dplyr v1.0.8,” “e1071 v1.7–9,” “earth v5.3.1,” “ggplot2 v3.3.5,” “pROC v1.18.0,” “dcurves0.4.0,” “Hmisc 5.1–0,” “PredictAbel 1.2–4,” “glmtoolbox 0.1.7,” “DescTools 0.99.49,” “randomForest v4.7–1,” and “xgboost v1.5.2.1” packages.

## 3 Results

### 3.1 Baseline Statistics

The final number of observations in the raw data set of 1937 patients was 1164 after cleaning. This consisted of 239 (20.5%) patients with cirrhosis and 925 (79.5%) patients without cirrhosis. When the variables were evaluated in general according to the presence or absence of cirrhosis, a homogeneous gender distribution was observed (*p* = 0.253). Apart from this, significance was noted in all variables. The basic statistics of the features in the cirrhosis groups are given in Table 1.

**TABLE 1:** Patient characteristics.

Features	No cirrhosis (*n* = 925)	Cirrhosis (*n* = 239)	*p*
AGE (years)	52 (36–63)	63 (55–70)	< 0.001
AST/ALT	0.9 (0.7–1.1)	0.9 (0.8–1.2)	< 0.001
TBIL (mg/dL)	0.6 (0.5–0.7)	0.7 (0.6–0.9)	< 0.001
Albumin (g/dL)	4.4 (4.2–4.5)	4.1 (3.9–4.3)	< 0.001
AFP (ng/ml)	3.2 (2.5–4.1)	6.6 (3.7–8.1)	< 0.001
ALP (U/L)	80.4 (77.3–87.5)	88 (79–105)	< 0.001
GGT (U/L)	33 (28.4–45.4)	56 (36–70)	< 0.001
Platelet (×10^3^/mL)	239.4 (222–259)	178 (134–229)	< 0.001
PT (seconds)	12.2 (12–12.6)	12.8 (12.2–13.6)	< 0.001
BMI (kg/m^2^)	25.5 (24.5–26.7)	26.8 (25.6–27.8)	< 0.001
CBC (×10^3^/mL)	7.5 (6.4–8.2)	6.4 (5.2–7.7)	< 0.001
APRI	0.4 (0.3–0.6)	0.8 (0.5–0.8)	< 0.001
FIB‐4	1.2 (0.8–1.7)	2.5 (1.8–2.8)	< 0.001
Gender			
F/M	430 (46.5)/495 (53.5)	121 (50.6)/118 (49.4)	0.253
Comorbidity			
No/Yes	567 (61.3)/358 (38.7)	87 (36.4)/152 (63.6)	< 0.001
CAD			
No/Yes	862 (93.2)/63 (6.8)	210 (87.9)/29 (12.1)	0.007
Hypertension			
No/Yes	704 (76.1)/221 (23.9)	129 (54)/110 (46)	< 0.001
DM			
No/Yes	810 (87.6)/115 (12.4)	175 (73.2)/64 (26.8)	< 0.001

*Note:* Numerical features were expressed as median (25th and 75th percentiles), and factors were expressed as frequency (percentage).

### 3.2 Evaluation of Models

The cirrhosis classification performances of the three ML techniques were evaluated in both the training and test sets. In the test set, the highest performance results (Accuracy = 0.89, Sen = 0.82, Spe = 0.92, AUC = 0.87, PPV = 0.90, NPV = 0.86) for 16 features were obtained from the RF method (hyperparameters; mtry = 4, ntree = 2500). The method with the lowest results (Accuracy = 0.84, Sen = 0.76, Spe = 0.91, AUC = 0.83, PPV = 0.88, NPV = 0.81) was the radial‐based SVM (hyperparameters; *c* = 2, Ɣ = 0.08). It was determined that the ML techniques provided approximately 87% accuracy in classifying cirrhosis with only the values obtained from routine laboratory tests and approximately 85% diagnosis (AUC) performance. In the training set, the highest performance results were obtained from the RF and xGBoost method (for both of them Accuracy = 1.00, Sen = 1.00, Spe = 1.00, AUC = 1.00, PPV = 1.00, NPV = 1.00). However, lower performance results were obtained from SVM with the radial basis function and linear kernel. Notably, the absence of machine learning models and the lack of hyperparameter optimization led to markedly inferior performance, comparable to random guessing (Table 2).

**TABLE 2:** Performance metrics of models.

Models (hyperparameters)	Metrics	Results	
		Training set	Test set
Random guessing	Accuracy	0.51 (0.48–0.55)	0.42 (0.38–0.46)
	AUC	0.55 (0.52–0.58)	0.54 (0.51–0.57)
	Sen	0.51 (0.48–0.54)	0.45 (0.40–0.50)
	Spe	0.51 (0.48–0.54)	0.37 (0.33–0.41)
	PPV	0.86 (0.82–0.91)	0.59 (0.55–0.63)
	NPV	0.15 (0.11–0.19)	0.27 (0.23–0.31)
RF (mtry = 4, ntree = 1500)	Accuracy	1.00 (0.99–1.00)	0.89 (0.85–0.93)
	AUC	1.00 (0.98–1.00)	0.87 (0.83–0.93)
	Sen	1.00 (0.99–1.00)	0.82 (0.77–0.84)
	Spe	1.00 (0.98–1.00)	0.92 (0.88–0.95)
	PPV	1.00 (0.99–1.00)	0.90 (0.86–0.93)
	NPV	1.00 (0.98–1.00)	0.86 (0.81–0.92)
XGBoost (nrounds = 340, max_depth = 4, eta = 0.3, gamma = 0, colsample_bytree = 0.8, min_child_weight = 1, subsample = 0.8)	Accuracy	1.00 (0.99–1.00)	0.87 (0.82–0.91)
	AUC	1.00 (0.97–1.00)	0.86 (0.83–0.93)
	Sen	1.00 (0.99–1.00)	0.79 (0.75–0.82)
	Spe	1.00 (0.98–1.00)	0.92 (0.88–0.95)
	PPV	1.00 (0.98–1.00)	0.90 (0.87–0.94)
	NPV	1.00 (0.99–1.00)	0.84 (0.80–0.90)
SVM‐linear (*c* = 0.28)	Accuracy	0.91 (0.87–0.95)	0.85 (0.83–0.90)
	AUC	0.90 (0.88–0.93)	0.83 (0.79–0.88)
	Sen	0.89 (0.86–0.93)	0.76 (0.70–0.79)
	Spe	0.92 (0.89–0.96)	0.92 (0.88–0.95)
	PPV	0.91 (0.88–0.94)	0.90 (0.87–0.94)
	NPV	0.90 (0.89–0.95)	0.81 (0.77–0.86)
**SVM‐Radial** (*c* = 2, Ɣ = 0.08)	Accuracy	0.90 (0.87–0.94)	0.84 (0.80–0.87)
	AUC	0.89 (0.85–0.93)	0.83 (0.78–0.88)
	Sen	0.89 (0.87–0.92)	0.76 (0.71–0.79)
	Spe	0.92 (0.89–0.98)	0.91 (0.87–0.95)
	PPV	0.91 (0.88–0.95)	0.88 (0.81–0.93)
	NPV	0.90 (0.87–0.93)	0.81 (0.77–0.86)

*Note:* Values in parentheses indicate a 95% confidence interval of estimates.

Abbreviations: AUC, area under the curve; NPV, negative predictive value; PPV, positive predictive value, Sen, sensitivity; Spe, specificity.

The diagnostic success of ML classification algorithms has been compared with the widely used APRI and FIB‐4. According to the ROC analysis results, even the radial‐based SVM with the lowest performance (AUC = 0.864) had higher diagnostic success than APRI (AUC = 0.779) and FIB‐4 (AUC = 0.841). Although the Brier scores for APRI (0.783) and FIB‐4 (0.761) show more favorable results, this score may indicate that the models are making overly confident predictions, potentially leading to overfitting, as these predictions often deviate from the true accuracy. Furthermore, other performance metrics, apart from the Brier score, demonstrate that ML methods perform better. The lowest chi‐square value (4.63 and *p* = 0.86) in terms of Hosmer–Lemeshow was obtained from the RF method. Positive and significant results were obtained for each ML method, with traditional APRI and FIB‐4 showing more favorable outcomes in both reclassification indices and discrimination ability. RF achieved a 53% improvement in classification performance compared to the APRI model. (*p* < 0.001). A 26% IDI value demonstrated that the RF model provided 26% better discrimination ability compared to the APRI (*p* < 0.001). Likewise, considering the FIB‐4 values, the performance results of the RF method were remarkable (NRI = 26%, *p* < 0.001 and IDI = 21%, *p* < 0.001) (Table 3).

**TABLE 3:** Comparison of APRI and FIB‐4 scores with ML techniques.

Overall performance	APRI	FIB‐4	SVM‐L	SVM‐R	RF	xGBoost
Brier Score	0.783	0.761	0.792	0.803	0.806	0.811
AUC	0.779	0.841	0.864	0.874	0.894	0.891
Calibration						
Hosmer–Lemeshow	29.6 (*p* < 0.001)	17.2 (*p* = 0.045)	6.12 (0.727)	33.4 (*p* < 0.001)	4.63 (*p* = 0.86)	5.88 (*p* = 0.75)
**Reclassification**	***APRI* vs *RF*** ***FIB‐4* vs *RF***	***APRI* vs *SVM‐L*** ***FIB‐4* vs *SVM‐L***	***APRI* vs *SVM‐R*** ***FIB‐4* vs *SVM‐R***		***APRI* vs *xGBoost*** ***FIB‐4* vs *xGBoost***	
NRI [95% CI]	0.53	0.47	0.46		0.41	
	[0.44–0.62]	[0.38–0.56]	[0.36–0.56]		[0.30–0.52]	
	0.26 [0.17–0.34]	0.20 [0.11–0.29]	0.21 [0.11–0.30]		0.15 [0.04–0.25]	
IDI [95% CI]	0.26	0.24	0.19		0.26	
	[0.22–0.31]	[0.19–0.28]	[0.15–0.24]		[0.21–0.32]	
	0.21 [0.15–0.26]	0.18 [0.13–0.23]	0.13 [0.09–0.18]		0.21 [0.15–0.26]	

*Note:* In the calculation of NRI, IDI, and Hosmer–Lemeshow values for RF, SVM, and xGBoost; it was obtained through the logistic model by using the predicted values obtained after the constructed model. The model created with APRI and FIB‐4 values was accepted as the old model, and the model created with ML techniques was accepted as the new model.

Abbreviations: SVM‐L, linear kernel SVM; SVM‐R, radial basis function SVM.

**FIGURE 4:**
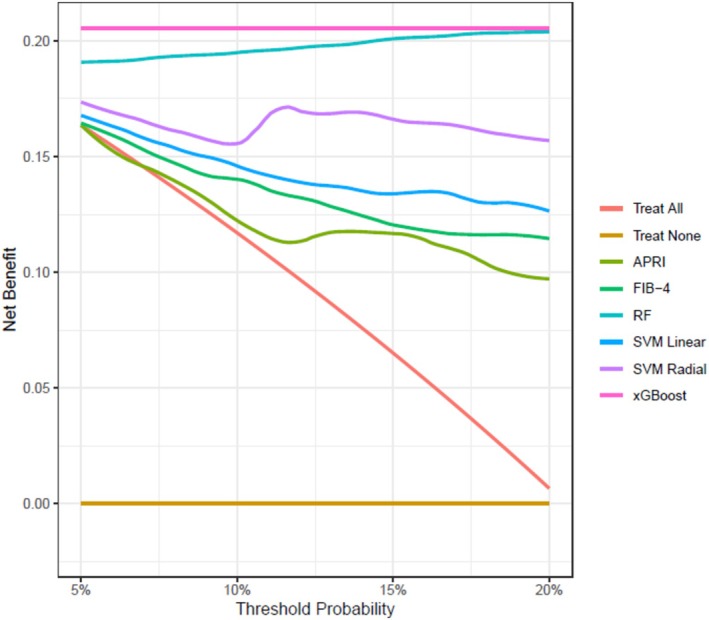
Decision curve analysis of values helpful in the diagnosis of cirrhosis.

Finally, when the success of the ML methods was analyzed with DCA, it was clearly seen that they were more more beneficial than the traditional APRI and FIB‐4 scores (Figure 4).

### 3.3 Important Features

**FIGURE 5:**
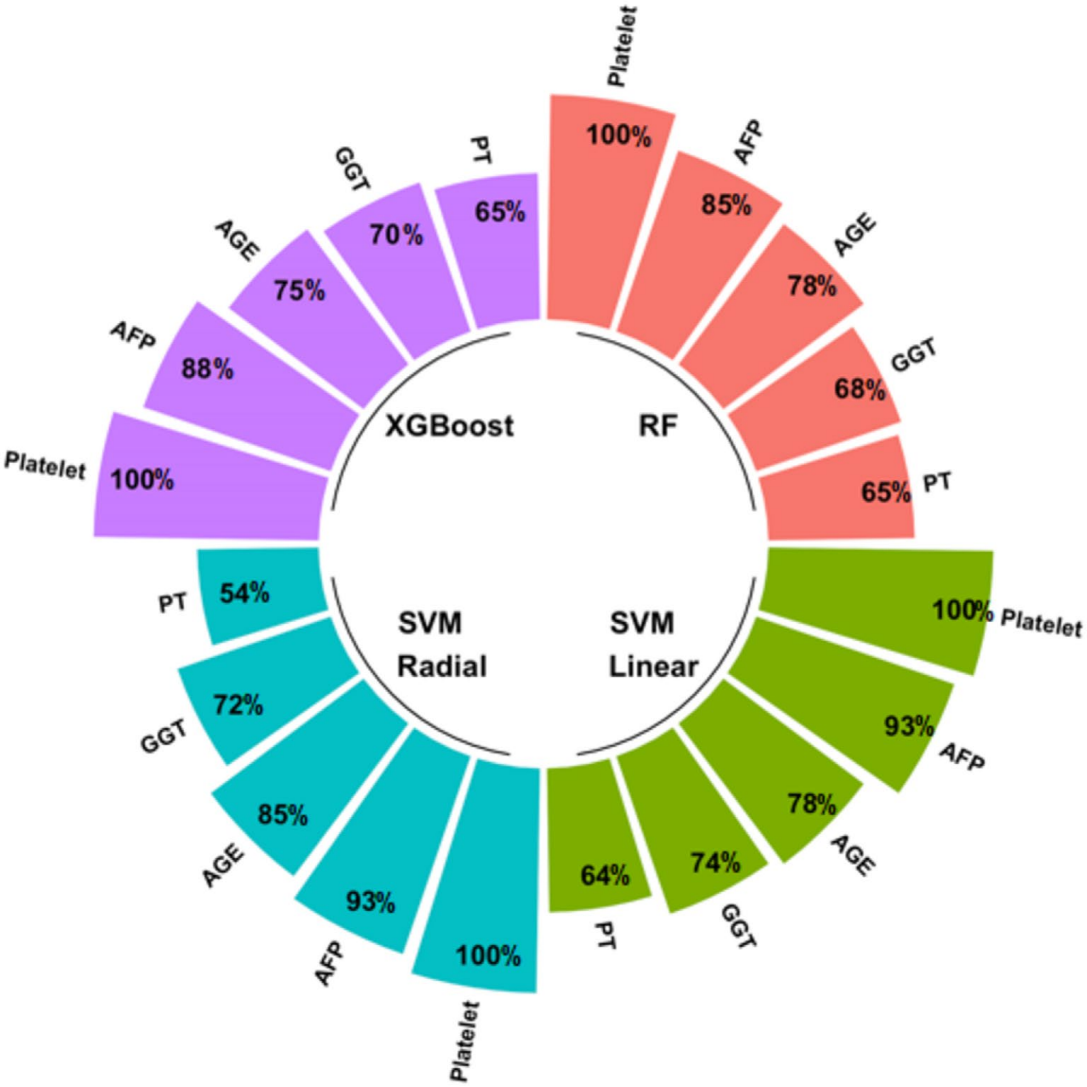
The five most important features in all ML algorithms.

The five most important features that contributed to the classification in all ML techniques were determined to be platelet (100%), AFP (85%–93%), age (68%–85%), GGT (70%–78%), and PT (54%–65%). Although the order of importance of age and GGT varies in the RF method, age was seen to be the third most important feature in general (Figure 5).

Optimum cut‐off values of these most important features to predict cirrhosis were found to be platelet: 182.000/mm^3^, AFP: 5.49 ng/mL, age: 52 years, GGT: 39.9 U/L, and PT: 12.35 s. With these cut‐off values, cirrhosis status was accepted as the outcome feature, and when univariate LR analysis was performed, the highest OR value AFP was obtained (COR = 9.11 (CI: 6.62–12.51), *p* < 0.001). As shown in Table 4, when the logistic model was adjusted considering all other features, the highest risk coefficient was obtained from platelet (AOR = 4.82 (CI: 3.13–7.41), *p* < 0.001).

**TABLE 4:** Model evaluations according to the cut‐off values of the most important features.

Features	Cut‐off value	COR (95% CI)	*p*	AOR (95% CI)	*p*
Platelet (×10^3^/mL)	< 182	8.76 (6.33–12.12)	< 0.001	4.82 (3.13–7.41)	< 0.001
AFP (ng/mL)	> 5.49	9.11 (6.62–12.51)	< 0.001	3.49 (2.32–5.27)	< 0.001
Age (years)	> 52	5.44 (3.77–7.84)	< 0.001	4.32 (2.67–6.97)	< 0.001
GGT (U/L)	> 39.9	5.48 (4.1–7.49)	< 0.001	3.04 (2.02–4.57)	< 0.001
PT (s)	> 12.35	4.02 (2.98–5.42)	< 0.001	2.20 (1.49–3.21)	< 0.001

*Note:* The cut‐off values were determined using ROC analysis, with the Youden Index maximizing both sensitivity and specificity for optimal classification performance.

Abbreviations: AOR, adjusted odds ratio; COR, Crude odds ratio.

## 4 Discussion

In this study, we focused on pre‐treatment CHC patients to ensure consistency and reliability in our predictive model. One of the main reasons for this choice is that our dataset consists of biopsy‐proven CHC cases. It is well established that liver pathophysiology undergoes significant changes following treatment, as numerous studies [29, 30, 31, 32] have demonstrated fibrosis and resolution of necroinflammation after viral eradication. Furthermore, including post‐treatment patients was avoided because machine learning models are trained on specific data distributions, and factors such as varying degrees of fibrosis in these patients could potentially affect the classification performance of the models. Thus, limiting the cohort to pre‐treatment cases ensures a more stable and interpretable model for clinicians.

Many ML classification algorithms (linear vector quantization (LVQ) logistic regression, naive Bayes, decision tree (DT), RF, XGBoost, K‐nearest neighbor, SVM, artificial neural networks (ANN), and classification and regression trees) have been used to evaluate and classify cirrhosis in different studies [5, 10, 11, 12, 13, 14]. Some of these studies used a data set consisting of quantitative data, such as the one we used in our study, and others used an ultrasonographic data set to predict cirrhosis. These studies have shown classification accuracy ranging from 80% to 97%. However, the mentioned studies generally did not go further than studies in which several ML techniques were applied to a data set, important features were determined, no details were made about treatments (especially DAA), and rather a kind of theoretical validation of ML methods was made. Regarding cirrhosis, studies that helped clinical decision‐makers or studies involving DAA therapy were usually studies in which cirrhosis was not classified and fibrosis stages were evaluated together [33, 34, 35, 36, 37]. For example, Emu et al. achieved an accuracy rate of > 97% with the three different ML algorithms (RF, MLP, and logarithmic regression), as well as Ghazal et al. with the SVM algorithm to predict the fibrosis stage. However, both of those studies used a UCI Egyptian data set [38] that included data on CHC patients receiving antiviral therapy [35, 39]. Failure to recognize the presence of cirrhosis before DAA therapy in CHC cases can lead to treatment failure and may also result in missing the chance of early detection and cure for HCC, which can be achieved with HCC surveillance [40].

APRI and FIB‐4 biochemical scores, which are the most widely used non‐invasive methods in clinical practice, have very low performance in predicting the presence of cirrhosis. As a matter of fact, in the study conducted by Parikh et al. [41] on 10,650 patients, the sensitivity and specificity of APRI at the time of cirrhosis diagnosis were found to be 9.3% and 98.8%, and those of FIB‐4 were 41.3% and 91.0%. In addition, the study conducted by Yen et al. on 1716 patients emphasized that the cut‐off values of APRI and FIB‐4 scores used in practice should be changed for the diagnosis of cirrhosis and that this change should be made according to AST values. In the mentioned study, scores with varying diagnostic performance were mentioned according to AST values (APRI's AUROC ranged from 0.81 to 0.68, and FIB‐4's ranged from 0.85 to 0.70) [42]. Therefore, it is not recommended to use these scores alone to predict advanced fibrosis or cirrhosis [7]. Consequently, there is a need for alternative non‐invasive methods. The results of the current study also showed that all ML models achieved a higher performance compared with APRI and FIB‐4 scores. Here's a concise explanation of the AOR and COR results for the discussion, tailored for hepatologists: These findings underscore the utility of these clinical markers in predicting cirrhosis, with AOR values providing more reliable estimates after controlling for confounding factors. The results suggest that these biomarkers, especially platelet count, AFP, and PT, should be carefully considered in clinical practice for cirrhosis risk stratification. These cut‐off values represent thresholds where clinical intervention might be considered based on the likelihood of an adverse outcome. The threshold probability, linked to DCA, helps in defining the level of certainty (probability) at which clinical action should be taken. By examining net benefit across varying threshold probabilities, clinicians can better understand how the model performs at different levels of risk tolerance. For instance, a higher threshold probability might indicate a greater certainty of treatment benefit, while a lower threshold could involve more inclusive treatment decisions for patients at moderate risk. Thus, the cut‐off values provide a practical and clinically relevant framework, offering hepatologists clear, actionable metrics based on the model's predictions. Additionally, DCA is helpful in assessing the net benefit at different thresholds, providing further insight into the clinical utility of the model across diverse risk profiles.

To date, it has been demonstrated that more successful results can be achieved in making sense of data with ML models. In addition, these modern techniques, which have many advantages compared to classical statistics, allow the determination of both complex relationships between features and the target variable and the most important features that affect the target variable [43, 44].

AFP, one of our most important attributes, is a glycoprotein secreted mainly from the fetal liver, and its main use in the monitoring of chronic liver diseases is HCC screening [17, 45]. However, it has been shown that serum AFP levels can often be high in CHC patients without HCC [46]. Although increased serum AFP levels are associated with increased levels of hepatic inflammation and fibrosis, which are highest in patients with cirrhosis, the clinical significance of elevated AFP in CHC patients has not been clearly demonstrated [47, 48]. Furthermore, results involving AFP in CHC patients diagnosed with cirrhosis are contradictory. According to Bayati et al. [49], AFP level > 17.8 ng/mL strongly suggests the diagnosis of cirrhosis in a population of patients with chronic hepatitis C, Fattovich et al. [50] reported that 43% of compensated cirrhotics had AFP levels 10 ng/mL, and Tong et al. [51] described AFP elevations in only 10% of cirrhotic patients. However, when Chyntia Olivia et al.'s [52] 10 ng/mL AFP cut‐off value for HCC is considered, the results create complete confusion. In the study conducted by Hashem et al., which included only a large number of patients, the cut‐off value of AFP was found to be > 6.5 ng/mL and was among the important features, but this value was a prediction for advanced fibrosis. Therefore, we think that the value of 5.49 for AFP in our study, which aims to diagnose cirrhosis patients with CHC only with a high number of patients and takes DAA therapy into account, is a very important auxiliary cut‐off for decision‐makers.

In the current study, platelet, age, GGT, and PT were other important features in the top five of all the models. In patients with chronic liver disease, thrombocytopenia is often the first presenting abnormality, seen in 6% of patients without cirrhosis, and in 70% of patients with cirrhosis [53]. In the current study, platelet was the most relevant feature, with a cut‐off value of < 182.000/mm^3^ for the prediction of cirrhosis with a 4.82‐fold increased risk. In the study conducted by Gordon et al. [54] on the non‐invasive diagnosis of cirrhosis with discriminant score in patients with CHC using the scoring method, they concluded that 0.74 sensitivity, 0.63 specificity, and 159,000/mm^3^ value of platelet were important. On the other hand, in the meta‐analysis conducted by Udell et al. [55], they calculated the negative likelihood ratio as 0.28 (0.07–0.48) for values below 200,000/mm^3^. As a general approach to the type of hepatitis, in the study of Surana et al. [56], they calculated the platelet value for the diagnosis of cirrhosis in chronic viral hepatitis B (HBV), C (HCV), and D (HDV) patients as 143,000/mm^3^. The values in the mentioned studies and similar studies in the literature focus on the values in the range of 140,000/mm^3^ and 200,000/mm^3^. Therefore, it was concluded that the 182,000/mm^3^ platelet value we recommended in our study was within the range and crucial.

Aging has been shown to lead to the impairment of hepatic functions and a reduction in liver regeneration and repair [57, 58]. Along with age, GGT and PT are included in many indices and are usually scored together with other variables. Therefore, it is very difficult to establish any consensus regarding the cut‐off values of each of these features. However, it is known that there is a positive relationship between the increase in these attributes and impairments in hepatic functions in almost all scoring used in the literature. The values we found in the current study showed that the relevance between these variables and cirrhosis was not different from the literature.

### 4.1 Limitations

There are some limitations in our study. First of all, since our study is aimed at the diagnosis of cirrhosis with CHC by considering DAA therapy, there is no equivalent in the literature. Therefore, this situation sometimes led us to comparisons with advanced fibrosis studies. In addition, while our model demonstrates strong predictive performance for pre‐treatment CHC patients, future research should explore its applicability to post‐treatment cases. Another limitation was that the number of cases was lower than in the other group. This led to a slight decrease in some performance metrics.

Taken together, these results revealed that the designed RF‐based ML model could diagnose cirrhosis with high accuracy using only routine laboratory test results. Although AFP is not included in non‐invasive indexes, it had a remarkable contribution in predicting cirrhosis, in addition to platelet, GGT, age, and PT features known to be important in predicting cirrhosis in CHC patients. Moreover, ML algorithms with the determined features had higher performance than APRI and FIB‐4 scores in assisting physicians in diagnosing cirrhosis.

## Ethics Statement

The authors have nothing to report.

## Conflicts of Interest

The authors declare no conflicts of interest.

## Supporting information


Table S1

## Data Availability

Research data are not shared.

## References

[1] Organization WH , “Global Progress Report on HIV, Viral Hepatitis and Sexually Transmitted Infections” (2021), https://www.who.int/publications/i/item/9789240027077.

[2] C. W. Spearman , G. M. Dusheiko , M. Hellard , and M. Sonderup , “Hepatitis C,” Lancet 394, no. 10207 (2019): 1451–1466, 10.1016/s0140-6736(19)32320-7. 31631857

[3] S. Lingala and M. G. Ghany , “Natural History of Hepatitis C,” Gastroenterology Clinics 44, no. 4 (2015): 717–734. 26600216 10.1016/j.gtc.2015.07.003 PMC5939344

[4] C.‐Y. Dai , W.‐L. Chuang , and M.‐L. Yu , “EASL Recommendations on Treatment of Hepatitis C: Final Update of the Series–Some Issues,” Journal of Hepatology 74, no. 2 (2021): 473–474. 33223214 10.1016/j.jhep.2020.10.013

[5] I. Hanif and M. M. Khan , “Liver Cirrhosis Prediction Using Machine Learning Approaches” (2022), IEEE:28‐34.

[6] T. S. Lim and J. K. Kim , “Is Liver Biopsy Still Useful in the Era of Non‐Invasive Tests?,” Clinical and Molecular Hepatology 26, no. 3 (2020): 302–304. 32646204 10.3350/cmh.2020.0081 PMC7364357

[7] L. Castera , H. Chan , M. Arrese , et al., “EASL‐ALEH Clinical Practice Guidelines: Non‐Invasive Tests for Evaluation of Liver Disease Severity and Prognosis,” Journal of Hepatology 63, no. 1 (2015): 237–264. 25911335 10.1016/j.jhep.2015.04.006

[8] K. Patel and G. Sebastiani , “Limitations of Non‐Invasive Tests for Assessment of Liver Fibrosis,” JHEP Reports 2, no. 2 (2020): 100067. 32118201 10.1016/j.jhepr.2020.100067 PMC7047178

[9] S. Jayatilake and G. U. Ganegoda , “Involvement of Machine Learning Tools in Healthcare Decision Making,” Journal of Healthcare Engineering 2021, no. 20 (2021): 6679512, 10.1155/2021/6679512. 33575021 PMC7857908

[10] K. Mala , V. Sadasivam , and S. Alagappan , “Neural Network Based Texture Analysis of CT Images for Fatty and Cirrhosis Liver Classification,” Applied Soft Computing 32 (2015): 80–86.

[11] O. M. Güneş , P. Kasap , and B. S. Çorba Zorlu , “The Comparison of Machine Learning Classification Algorithms Used to Diagnose Liver Cirrhosis Disease and a Brief Review,” Concurrency and Computation: Practice and Experience 35, no. 8 (2023): e7628.

[12] A. Bedeir and M. El‐Had , “A Proposed Framework for Predictive Analytics for Cirrhosis of the Liver Using Machine Learning,” Journal of the Egyptian Society for Information Systems and Computer Technology 31, no. 31 (2023): 114–123.

[13] Y. Cao , Z. D. Hu , X. F. Liu , A. M. Deng , and C. J. Hu , “An MLP Classifier for Prediction of HBV‐Induced Liver Cirrhosis Using Routinely Available Clinical Parameters,” Disease Markers 35, no. 6 (2013): 653–660, 10.1155/2013/127962. 24302810 PMC3834663

[14] A. Utku , “Deep Learning Based Cirrhosis Detection,” Operational Research in Engineering Sciences: Theory and Applications 6, no. 1 (2023): 95–114.

[15] T. Poynard , P. Bedossa , and P. Opolon , “Natural History of Liver Fibrosis Progression in Patients With Chronic Hepatitis C,” Lancet 349, no. 9055 (1997): 825–832. 9121257 10.1016/s0140-6736(96)07642-8

[16] C. T. Wai , J. K. Greenson , R. J. Fontana , et al., “A Simple Noninvasive Index Can Predict Both Significant Fibrosis and Cirrhosis in Patients With Chronic Hepatitis C,” Hepatology 38, no. 2 (2003): 518–526, 10.1053/jhep.2003.50346. 12883497

[17] R. K. Sterling , E. Lissen , N. Clumeck , et al., “Development of a Simple Noninvasive Index to Predict Significant Fibrosis in Patients With HIV/HCV Coinfection,” Hepatology 43, no. 6 (2006): 1317–1325, 10.1002/hep.21178. 16729309

[18] M. A. M. Hasan , M. Nasser , S. Ahmad , and K. I. Molla , “Feature Selection for Intrusion Detection Using Random Forest,” Journal of Information Security 7, no. 3 (2016): 129–140.

[19] C. Ray and A. Ray , “Intrapartum Cardiotocography and Its Correlation With Umbilical Cord Blood pH in Term Pregnancies: A Prospective Study,” International Journal of Reproduction, Contraception, Obstetrics and Gynecology 6, no. 7 (2017): 2745–2753.

[20] A. A. Soofi and A. Awan , “Classification Techniques in Machine Learning: Applications and Issues,” Journal of Basic and Applied Sciences 13 (2017): 459–465.

[21] V. F. Rodriguez‐Galiano , B. Ghimire , J. Rogan , M. Chica‐Olmo , and J. P. Rigol‐Sanchez , “An Assessment of the Effectiveness of a Random Forest Classifier for Land‐Cover Classification,” ISPRS Journal of Photogrammetry and Remote Sensing 67 (2012): 93–104.

[22] A. Mathur and G. M. Foody , “Multiclass and Binary SVM Classification: Implications for Training and Classification Users,” IEEE Geoscience and Remote Sensing Letters 5, no. 2 (2008): 241–245.

[23] L. Torlay , M. Perrone‐Bertolotti , E. Thomas , and M. Baciu , “Machine Learning–XGBoost Analysis of Language Networks to Classify Patients With Epilepsy,” Brain Informatics 4, no. 3 (2017): 159–169. 28434153 10.1007/s40708-017-0065-7 PMC5563301

[24] J. A. Sidey‐Gibbons and C. J. Sidey‐Gibbons , “Machine Learning in Medicine: A Practical Introduction,” BMC Medical Research Methodology 19, no. 1 (2019): 1–18. 30890124 10.1186/s12874-019-0681-4 PMC6425557

[25] D. W. Hosmer , S. Lemeshow , and J. Klar , “Goodness‐of‐Fit Testing for the Logistic Regression Model When the Estimated Probabilities Are Small,” Biometrical Journal 30, no. 8 (1988): 911–924.

[26] M. J. Pencina , R. B. D'Agostino Sr , R. B. D'Agostino Jr , and R. S. Vasan , “Evaluating the Added Predictive Ability of a New Marker: From Area Under the ROC Curve to Reclassification and Beyond,” Statistics in Medicine 27, no. 2 (2008): 157–172. 17569110 10.1002/sim.2929

[27] M. J. Pencina , R. B. D'Agostino, Sr. , and E. W. Steyerberg , “Extensions of Net Reclassification Improvement Calculations to Measure Usefulness of New Biomarkers,” Statistics in Medicine 30, no. 1 (2011): 11–21. 21204120 10.1002/sim.4085 PMC3341973

[28] A. J. Vickers and E. B. Elkin , “Decision Curve Analysis: A Novel Method for Evaluating Prediction Models,” Medical Decision Making 26, no. 6 (2006): 565–574. 17099194 10.1177/0272989X06295361 PMC2577036

[29] W. S. Oh , S. T. Heo , S. H. Kim , W. J. Choi , M. G. Han , and J. Y. Kim , “Plasma Exchange and Ribavirin for Rapidly Progressive Severe Fever With Thrombocytopenia Syndrome,” International Journal of Infectious Diseases 18 (2014): 84–86. 24161209 10.1016/j.ijid.2013.08.011

[30] T. Poynard , V. Ratziu , F. Charlotte , Z. Goodman , J. McHutchison , and J. Albrecht , “Rates and Risk Factors of Liver Fibrosis Progression in Patients With Chronic Hepatitis C,” Journal of Hepatology 34, no. 5 (2001): 730–739. 11434620 10.1016/s0168-8278(00)00097-0

[31] M. Enomoto , Y. Ikura , A. Tamori , et al., “Short‐Term Histological Evaluations After Achieving a Sustained Virologic Response to Direct‐Acting Antiviral Treatment for Chronic Hepatitis C,” United European Gastroenterology Journal 6, no. 9 (2018): 1391–1400. 30386612 10.1177/2050640618791053 PMC6206535

[32] D. C. Rockey and S. L. Friedman , “Fibrosis Regression After Eradication of Hepatitis C Virus: From Bench to Bedside,” Gastroenterology 160, no. 5 (2021): 1502–1520. e1. 33529675 10.1053/j.gastro.2020.09.065 PMC8601597

[33] M. B. Butt , M. Alfayad , S. Saqib , et al., “Diagnosing the Stage of Hepatitis C Using Machine Learning,” Journal of Healthcare Engineering 2021 (2021): 8062410. 35028114 10.1155/2021/8062410 PMC8748759

[34] M. Emu , F. B. Kamal , S. Choudhury , and T. E. A. de Oliveira , “Assisting the Non‐Invasive Diagnosis of Liver Fibrosis Stages Using Machine Learning Methods,” Annual International Conference of the IEEE Engineering in Medicine and Biology Society 2020 (2020): 5382–5387. 33019198 10.1109/EMBC44109.2020.9176542

[35] T. M. Ghazal , M. Anam , M. K. Hasan , et al., “Hep‐Pred: Hepatitis C Staging Prediction Using Fine Gaussian SVM,” Computational Materials Continua 69, no. 1 (2021): 191–203, 10.32604/cmc.2021.015436.

[36] A. Akella and S. Akella , “Applying Machine Learning to Evaluate for Fibrosis in Chronic Hepatitis C” (2020), medRxiv.

[37] A. Hashem , A. Awad , H. Shousha , et al., “Validation of a Machine Learning Approach Using FIB‐4 and APRI Scores Assessed by the Metavir Scoring System: A Cohort Study,” Arab Journal of Gastroenterology 22, no. 2 (2021): 88–92. 33985905 10.1016/j.ajg.2021.04.003

[38] G. Shiha , R. Soliman , N. N. Mikhail , et al., “Development and Multicenter Validation of FIB‐6: A Novel, Machine Learning, Simple Bedside Score to Rule out Liver Cirrhosis and Compensated Advanced Chronic Liver Disease in Patients With Chronic Hepatitis C,” Hepatology Research 52, no. 2 (2022): 165–175. 34767312 10.1111/hepr.13729

[39] S. C. Nandipati , C. XinYing , and K. K. Wah , “Hepatitis C Virus (HCV) Prediction by Machine Learning Techniques,” Applications of Modelling and Simulation 4 (2020): 89–100.

[40] D. Jain and V. Singh , “Feature Selection and Classification Systems for Chronic Disease Prediction: A Review,” Egyptian Informatics Journal 19, no. 3 (2018): 179–189.

[41] N. D. Parikh , M. Mehta , and E. B. Tapper , “FIB‐4 and APRI for Cirrhosis Detection in a Privately Insured National Cohort,” JHEP Reports: Innovation in Hepatology 6, no. 1 (2024): 100925, 10.1016/j.jhepr.2023.100925. 38074510 PMC10701128

[42] Y. H. Yen , F. Y. Kuo , K. M. Kee , et al., “APRI and FIB‐4 in the Evaluation of Liver Fibrosis in Chronic Hepatitis C Patients Stratified by AST Level,” PLoS One 13, no. 6 (2018): e0199760, 10.1371/journal.pone.0199760. 29953518 PMC6023204

[43] A. Dhillon and A. Singh , “Machine Learning in Healthcare Data Analysis: A Survey,” Journal of Biology and Today's World 8, no. 6 (2019): 1–10.

[44] Z. Barnett‐Itzhaki , M. Elbaz , R. Butterman , et al., “Machine Learning vs. Classic Statistics for the Prediction of IVF Outcomes,” Journal of Assisted Reproduction and Genetics 37, no. 10 (2020): 2405–2412, 10.1007/s10815-020-01908-1. 32783138 PMC7550518

[45] A. Vallet‐Pichard , V. Mallet , B. Nalpas , et al., “FIB‐4: An Inexpensive and Accurate Marker of Fibrosis in HCV Infection. Comparison With Liver Biopsy and Fibrotest,” Hepatology 46, no. 1 (2007): 32–36, 10.1002/hep.21669. 17567829

[46] T. Isac , S. Isac , S. Ioanitescu , et al., “Dynamics of Serum α‐Fetoprotein in Viral Hepatitis C Without Hepatocellular Carcinoma,” Experimental and Therapeutic Medicine 22, no. 1 (2021): 749. 34035846 10.3892/etm.2021.10181 PMC8135122

[47] H. Dabbous , R. El‐Folly , H. EL‐Nakeeb , A. Helmy , and S. Saleh , “Evaluation of the Role of Alpha‐Fetoprotein (AFP) Levels in Chronic Viral Hepatitis C Patients, Wit‐Hout Hepatocellular Carcinoma (HCC),” Journal of Medical Science and Clinical Research 3, no. 3 (2015): 4787–4800.

[48] A. M. Di Bisceglie , R. K. Sterling , R. T. Chung , et al., “Serum Alpha‐Fetoprotein Levels in Patients With Advanced Hepatitis C: Results From the HALT‐C Trial,” Journal of Hepatology 43, no. 3 (2005): 434–441. 16136646 10.1016/j.jhep.2005.03.019

[49] N. Bayati , A. L. Silverman , and S. C. Gordon , “Serum Alpha‐Fetoprotein Levels and Liver Histology in Patients With Chronic Hepatitis C,” American Journal of Gastroenterology 93, no. 12 (1998): 2452–2456. 9860408 10.1111/j.1572-0241.1998.00703.x

[50] G. Fattovich , D. F. GiustinaG , F. Degos , et al., “Morbidity and Mortality in Compensated Cirrhosis Type C: A Retrospective Follow‐Up Study of 384 Patients,” Gastroenterology 112 (1997): 463–472, 10.1053/gast.1997.v112.pm9024300. 9024300

[51] M. J. Tong , N. S. El‐Farra , A. R. Reikes , and R. L. Co , “Clinical Outcomes After Transfusion‐Associated Hepatitis C,” New England Journal of Medicine 332, no. 22 (1995): 1463–1466. 7739682 10.1056/NEJM199506013322202

[52] C. O. M. Jasirwan , A. Fahira , L. Siregar , and I. Loho , “The Alpha‐Fetoprotein Serum Is Still Reliable as a Biomarker for the Surveillance of Hepatocellular Carcinoma in Indonesia,” BMC Gastroenterology 20, no. 1 (2020): 215, 10.1186/s12876-020-01365-1. 32646378 PMC7346661

[53] A. H. Moore , “Thrombocytopenia in Cirrhosis: A Review of Pathophysiology and Management Options,” Clinical Liver Disease 14, no. 5 (2019): 183–186. 31879561 10.1002/cld.860 PMC6924969

[54] A. Gordon , M. J. Bailey , P. R. Gibson , and S. K. Roberts , “Comprehensive Clinical Assessment Improves the Accuracy of Predicting Cirrhosis in Chronic Hepatitis C,” Journal of Gastroenterology and Hepatology 20, no. 6 (2005): 825–832, 10.1111/j.1440-1746.2005.03828.x. 15946128

[55] J. A. Udell , C. S. Wang , J. Tinmouth , et al., “Does This Patient With Liver Disease Have Cirrhosis?,” Journal of the American Medical Association 307, no. 8 (2012): 832–842. 22357834 10.1001/jama.2012.186

[56] P. Surana , J. Hercun , V. Takyar , D. E. Kleiner , T. Heller , and C. Koh , “Platelet Count as a Screening Tool for Compensated Cirrhosis in Chronic Viral Hepatitis,” World Journal of Gastrointestinal Pathophysiology 12, no. 3 (2021): 40–50, 10.4291/wjgp.v12.i3.40. 34084591 PMC8160599

[57] N. J. Hunt , S. W. S. Kang , G. P. Lockwood , D. G. Le Couteur , and V. C. Cogger , “Hallmarks of Aging in the Liver,” Computational and Structural Biotechnology Journal 17 (2019): 1151–1161, 10.1016/j.csbj.2019.07.021. 31462971 PMC6709368

[58] N. A. Timchenko , “Aging and Liver Regeneration,” Trends in Endocrinology and Metabolism 20, no. 4 (2009): 171–176. 19359195 10.1016/j.tem.2009.01.005

