# Supplementary material for: Assisting the Diagnosis of Cirrhosis in Chronic Hepatitis C Patients Based on Machine Learning Algorithms: A Novel Non‐Invasive Approach
Source: J Clin Lab Anal. 2025 May 19;39(12):e70054. doi: 10.1002/jcla.70054 (PMC12179807; doi:10.1002/jcla.70054)
Supplement: Supplementary file 1 — Table S1 [file JCLA-39-e70054-s001.docx]

| **Supplementary Table 1** Accuracy of ML models with all data | | | |
| --- | --- | --- | --- |
| **Models** | **Metric** | **Results** | |
|  |  | **Training Set** | **Test Set** |
| **RF** | Accuracy | 0.82 (0.76 – 0.86) | 0.76 (0.70 - 0.84) |
| **XGBoost** |  | 0.84 (0.80 - 0.85) | 0.81 (0.78 - 0.85) |
| **SVM-Linear** |  | 0.91 (0.88 - 0.95) | 0.70 (0.63 - 0.75) |
| **SVM-Radial** |  | 0.87 (0.84 - 0.91) | 0.67 (0.80 - 0.87) |
| Accuracy values obtained before removing outliers and noisy data from the data set and performance differences between models reveal the indispensability of pre-processing the data. Hyperparameters were determined with the values in the models that give the highest performance. | | | |
